# Emergence of *Escherichia coli* sequence type 131 clone carrying *bla*_CTX-M_ genes in Guilan Province, Northern Iran

**DOI:** 10.1128/spectrum.02270-24

**Published:** 2025-08-19

**Authors:** Samaneh Kazemi, Ali Mojtahedi, Farzaneh Hosseini

**Affiliations:** 1 Department of Microbiology, NT. C., Islamic Azad University 68106 , Tehran, Iran; 2 Department of Microbiology, School of Medicine, Iran University of Medical Sciences 440827, Tehran, Iran; University of Brescia, Brescia, Italy

**Keywords:** antibiotic resistance, *Escherichia coli*, beta-lactamase CTX-M, urinary tract infections

## Abstract

Extended-spectrum beta-lactamase (ESBL)-producing Enterobacterales complicate clinical management, as they are resistant to standard antibiotics and often necessitate intricate treatment plans. As a result, continuous monitoring is essential to prevent the spread of resistant strains and ensure effective treatment options are available. Enterobacterales are humans’ primary contributors to urinary tract infections (UTIs). Among these, *Escherichia coli* (*E. coli*) sequence type 131 (ST131), which carries β-lactamase genes with significant virulence potential, has been documented globally. *E. coli* ST131 mostly belongs to the serotypes O25 and O16 and is a well-defined subclone of the FimH30 and FimH30-Rx. We aimed to evaluate the impact of the ST131 clone carrying three β-lactamase genes—the *bla*_CTX-M-1_, *bla*_CTX-M-14_, and *bla*_CTX-M-15_ genes—in the antibiotic resistance pattern of ESBL-producing *E. coli* (ESBL-EC) from UTIs in Guilan, Iran. The phenotypic confirmatory disc diffusion test (PCDDT), according to CLSI recommendations, was utilized to detect ESBL production. The ESBL-EC isolates were screened for the ST131 clone using specific PCR of O25b, O16, H30, and H30-Rx. Out of the 107 ESBL-EC, 70 (65.4%) showed as ST131. Among the ST131 clones, the highest frequency belonged to the ST131-H30 (98.6%). Among the beta-lactam antibiotics, both the ST131 and non-ST131 groups exhibited complete resistance (100%) to cefotaxime, ampicillin, amoxicillin-clavulanate, cephalothin, cefixime, and ceftriaxone while demonstrating the greatest susceptibility to imipenem (*R* = 0%). In the case of non-beta-lactam antibiotics, both groups showed significant resistance (approximately 91%) to nalidixic acid, while displaying the highest susceptibility to nitrofurantoin (up to 83%). Our study confirmed the dominance of the ST131 clone among the ESBL-EC and β-lactamase genes associated with the transmission of this pandemic clone.

**IMPORTANCE:**

Various antibiotic resistance and virulence genes highlight challenges in clinical management and emphasize the need for effective treatment strategies to reduce the risks associated with UTIs. Continuous research is essential to improve knowledge and create innovative approaches to tackle these urological conditions. Our study confirmed the predominance of genes related to the ST131 clone, including O25, O16, H30, and H30-Rx, among ESBL-EC. We found that the *E. coli* ST131 clone carried *bla*_CTX-M-1_, *bla*_CTX-M-14_, and *bla*_CTX-M-15_ and was expanded in our locale. The clones differed significantly in resistance, susceptibility, and relative susceptibility, only in antibiotic resistance in the case of nitrofurantoin (*P* < 0.001). Combining resistance genes in the ST131 clone can facilitate its rapid and successful global spread.

## INTRODUCTION

*Escherichia coli (E. coli*) is a common microorganism found in the intestinal microbiota of humans and animals. It can act as both an opportunistic and zoonotic pathogen affecting both humans and animals (1). Globally, *E. coli* that produces extended-spectrum beta-lactamase (ESBL) is ubiquitous. The globally dominant sequence type (ST)131 clone is the most prevalent genotype (2). A significant global public health concern is the emergence of *E. coli* ST131, a strain that is both virulent and resistant to multiple antibiotics. Various international studies have shown the widespread distribution of this clone; in some regions, ST131 accounts for as much as 30% of all *E. coli* isolates (3). Whole-genome sequencing (WGS) has been used in several ST131 research studies. Since the strains in ST131 are closely related and seem to have a common origin, the strains are frequently referred to as clones or clonal groups. Using two closely comparable naming approaches, the ST131 strains were further classified into three primary groups: A/H41, B/H22, and C/H30-R (4). H30 is associated with allele 30 of the type 1 fimbrial adhesin FimH. FimH promotes the colonization and invasion of the bladder epithelium by bacteria, forms biofilms, binds to intestinal crypts, and helps establish a stable reservoir in the intestines (2, 4). The FimH30 subclones have spread globally as a result of clonal expansion (5). Further studies indicated that most ESBL-producing multidrug-resistant (MDR) ST131 isolates were also part of the subclonal H30-R group (6–8). The WGS analysis identified H30-Rx as a specific clonal subgroup within the subclonal H30-R group (8, 9). Although the O25 serotype constitutes the majority of ST131 isolates, a small number of O16 serotype ST131 isolates have recently been detected in several countries (10–16).

The prevalence and epidemiology of ESBL-producing *E. coli* are changing very rapidly. In recent years, ESBL production in *E. coli* has significantly increased primarily due to the spread of CTX-M β-lactamases (Cefotaximases) (12). The *bla*_CTX-M-1_ and *bla*_CTX-M-14_ enzymes are the most common ESBL in *E. coli* isolated from livestock, especially poultry and foods of animal origin (17, 18). The *bla*_CTX-M-14_ is the second most prevalent ESBL type globally (19), caused by both ST131 and non-ST131 *E. coli* (20–23). Nevertheless, the *bla*_CTX-M-15_ enzyme is the most widely used in clinical settings and, with few exceptions, in the public worldwide (24).

Urinary tract infections (UTIs) are among the most prevalent infectious diseases. They are common in all age groups and can be either hospital-acquired (nosocomial) or acquired in the community. UTIs are especially significant in the elderly, leading to considerable morbidity for both outpatients and hospitalized patients. Uropathogenic *E. coli* (UPEC) strains are the leading causes of UTIs. Over 90% of these infections are acquired in the community, while the remainder are hospital-acquired UTIs. The incidence of multidrug-resistant *E. coli*, capable of producing extended-spectrum β-lactamases (ESBL-MDR-EC), is increasing worldwide (25–29). Most investigations regarding the distribution of ST131 among *E. coli* isolates causing UTIs have focused on ESBL-producing isolates (30–32). We aimed to evaluate the impact of the ST131 clone carrying three β-lactamase genes—the *bla*_CTX-M-1_, *bla*_CTX-M-14_, and *bla*_CTX-M-15_ genes—on the antibiotic resistance pattern of ESBL-producing *E. coli* (ESBL-EC) from UTIs in Guilan, Iran.

## MATERIALS AND METHODS

### Study design and bacterial isolates

**Fig 1:**
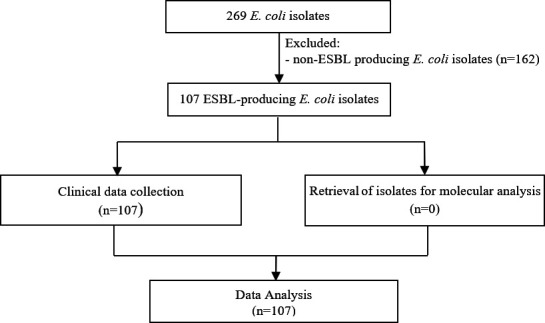
Flow chart of study samples. A flow chart for study enrollment and reasons for sample exclusion is shown.

This descriptive cross-sectional study utilized 269 *E. coli* isolates obtained from urine samples of hospitalized patients in Rasht, Iran. Following the phenotypic confirmatory disc diffusion test (PCDDT), 162 strains were not found to be ESBL-EC and were thrown out. Thus, the final analyzed set consisted of 107 strains (Fig. 1).

### Screening of ESBL phenotype

We studied ESBL-EC isolates using PCDDT with cefotaxime-clavulanic acid and ceftazidime-clavulanic acid as a two-disc synergy compared to ceftazidime and cefotaxime alone. The increased inhibition zone of ≥5 mm around the intervertebral discs with clavulanic acid indicates that the bacterium is ESBL-producing (33).

### Antimicrobial susceptibility testing

Mast (Merseyside, UK) antibiotic discs were used to test the antimicrobial susceptibility of ESBL-EC isolates according to the Clinical and Laboratory Standards Institute (CLSI-2020-M100-S30) (34). The antibiotics used for the disc diffusion method were included (µg/disc): cefotaxime (30), ampicillin (10), ciprofloxacin (5), cefoxitin (30), aztreonam (30), amoxicillin-clavulanate (30), tetracycline (30), ofloxacin (5), cephalothin (30), gentamicin (120), nalidixic acid (30), trimethoprim/sulfamethoxazole (1.25/23.75) (co-trimoxazole [25]), cefixime (5), nitrofurantoin (300), imipenem (10), ceftriaxone (30), and ceftazidime (300).

To control the accuracy and correctness of the antibiotic discs, the standard strain was used to control the quality of the used discs while performing the test on the patient samples. *E. coli* PTCC No. 1399 (ATCC 25922), obtained from the Iran Research Organization for Science and Technology (IROST), was utilized as a standard strain.

### Genomic DNA extraction

Plasmid DNA extraction from fresh ESBL-EC isolates was done using the Gen JET Plasmid Miniprep Kit (Fermentas, Lithuania) according to the manufacturer’s instructions.

### Molecular detection by polymerase chain reaction (PCR) and electrophoresis

Carriage of β-lactamase genes associated with three β-lactamase genes (*bla*_CTX-M-1_, *bla*_CTX-M-14_, and *bla*_CTX-M-15_) and genes linked to the ST131 clone (O25, O16, H30, and H30-Rx) was detected by specific primers using PCR (8, 35–40). Germany’s Metabion Co. provided the primers. Data from the primers used in this study are shown in Table 1. The PCR products were separated on 1.5% agarose gel and visualized by ultraviolet light.

**TABLE 1:** Characteristics of primers used in this study

Region detected	Primer designation	Primer sequences (5′→3′)	Protein function	Size of product (bp)	Ref.
CTX-M-1	CTX-M-F	TTT GCG ATG TGC AGT ACC AGT AA	Extended-spectrum β-lactamase	543	(35)
	CTX-M-R	CGA TAT CGT TGG TGG TGC CAT A			
CTX-M-14	CTX-M-14-F	CTG ATG TAA CAC GGA TTG ACC	Extended-spectrum β-lactamase	871	(36)
	CTX-M-14-R	CGA TTT ATT CAA CAA AAC CAG			
CTX-M-15	CTX-M-15-F	AGA ATA AGG AAT CCC ATG GTT	Extended-spectrum β-lactamase	875	(37)
	CTX-M-15-R	ACC GTC GGT GAC GAT TTT AG			
O25b-ST131	O25b-ST131-F	TCC AGC AGG TGC TGG ATC GT	O-antigen flippase	347	(38)
	O25b-ST131-R	GCG AAA TTT TTC GCC GTA CTG T			
O16-ST131	O16-ST131-F	AAA ACC GCG CCG CGT TAC CT	O-antigen polymerase	145	(39)
	O16-ST131-R	CCA GAA ATC GCG CCC GCA TT			
ST131- H30	ST131-H30-F	CCG CCA ATG GTA CCG CTA TT	Flagellin	354	(40)
	ST131-H30-R	CAG CTT TAA TCG CCA CCC CA			
ST131- H30-Rx	ST131-H30-Rx-F	GGT TGC GGT CTG GGC A	Flagellin	194	(8)
	ST131-H30-Rx-R	CAA TAT CCA GCA CGT TCC AGG TG			

### DNA sequencing

The PCR product was subjected to sequencing (Topazgene Co., Alborz, Iran). Subsequently, the sequences were confirmed by comparison with available sequences in the NCBI database (http://www.ncbi.nlm.nih.gov/BLAST/).

### Statistical analysis

The χ squared (*X*^2^) and Fisher exact tests were used to analyze the prevalence of antibiotic resistance genes between the ST131 and non-ST131 isolates to determine correlation. All tests were performed using SPSS version 26. *P* ≤ 0.05 was considered statistically significant.

## RESULTS

### Clinical features

Of the 269 *E. coli* isolates, 107 (39.8%) were ESBL-EC. Of the 107 ESBL-EC isolates, 67 samples belonged to women (62.6%) and 40 to men (37.4%). Among these, 70 (65.4%) isolates were identified as ST131. The relative frequency distribution of ESBL-EC strains by sex, separated into ST131 and non-ST131, is shown in Table 2. According to Fisher’s exact test, no significant difference was observed between ESBL-EC ST131 and non-ST131 groups by sex (*P* = 0.677).

**TABLE 2:** Relative frequency distribution of ESBL-producing *Escherichia coli* strains (ST131 and non-ST131) isolated from hospitalized patients with urinary tract infections according to sex

Sex	ESBL–ST131 (*N* = 70)		ESBL–non-ST131 (*N* = 37)	
	No.	%	No.	%
Female	45	64.3	22	59.5
Male	25	35.7	15	40.5

According to the Kolmogorov–Smirnov test, the quantitative variable age did not have a normal distribution (*P* < 0.001). The median age of the subjects whose ESBL-EC strains were isolated from their urine samples was set at 55 years, and their interquartile range (IQR) was 35 to 71 years. Additionally, the youngest person was one month old, and the oldest person was 87 years old. Of the 107 ESBL-EC strains, 43 (40.2%) with the highest frequency belonged to the age group > 61 years (Table 3). Even for ST131 and non-ST131 strains, the highest frequency belongs to the same age group. Based on Fisher’s exact test, no significant difference was observed between the ESBL-EC ST131 and non-ST131 strains depending on age group (*P* = 0.908).

**TABLE 3:** Relative frequency distribution of ESBL-producing *Escherichia coli* strains isolated from hospitalized patients with urinary tract infections according to age groups

Age (years)	ESBL–EC (*N* = 107)	
	No.	%
<1	6	5.6
1–15	11	10.3
16–30	7	6.5
31–45	13	12.1
46–60	27	25.2
>61	43	40.2

### Prevalence of ST131 clone

Among the ST131 clones, the highest frequency belonged to the ST131- H30 (98.6%), and the lowest frequency belonged to the O16-ST131 (77.1%). At least three and at most four genes were present in all ST131 strains. In 65.7% of the strains, all four genes, including O25b-ST131, O16-ST131, ST131- H30, and ST131- H30-Rx, were observed simultaneously (Table 4).

**TABLE 4:** Frequency distribution of different ST131 subclones (*N* = 70) in ESBL-producing *Escherichia coli* strains isolated from hospitalized patients with urinary tract infections

Gene	Gene presence	No.	%
O25b-ST131	Positive	68	97.1
O16-ST131	Positive	54	77.1
ST131- H30	Positive	69	98.6
ST131- H30-Rx	Positive	65	92.9
O25b-ST131, O16-ST131, ST131- H30	All three positive	5	7.1
O25b-ST131, ST131- H30, ST131- H30-Rx	All three positive	16	22.8
O25b-ST131, O16-ST131, ST131- H30-Rx	All three positive	1	1.4
O16-ST131, ST131- H30, ST131- H30-Rx	All three positive	2	2.8
O25b-ST131, O16-ST131, ST131- H30, ST131- H30-Rx	All four positive	46	65.7

### Prevalence of β-lactamase genes

The relative frequency of *bla*_CTX-M-14_ and *bla*_CTX-M-15_ genes in ESBL-EC strains is 86.9%. Furthermore, the relative frequency of these two genes between ST131 and non-ST131 strains averaged more than 85%. ESBL-EC strains ST131 and non-ST131 did not have a significant statistical association between the frequencies of *bla*_CTX-M-1_, *bla*_CTX-M-14_, and *bla*_CTX-M-15_ genes, as shown by Fisher’s exact test (Table 5).

**TABLE 5:** Relative frequency distribution of *bla*_CTX-M_, *bla*_CTX-M-14_, and *bla*_CTX-M-15_ genes in ESBL-producing *Escherichia coli* strains (ST131 and non-ST131) isolated from hospitalized patients with urinary tract infections

Gene	ESBL (*N* = 107)		ESBL–ST131 (*N* = 70)		ESBL–non-ST131 (*N* = 37)		*P*-value
	No.	%	No.	%	No.	%	
*bla*_CTX-M-1_	87	81.3	57	81.4	30	81.1	1.000
*bla*_CTX-M-14_	93	86.9	61	87.1	32	86.5	1.000
*bla*_CTX-M-15_	93	86.9	60	85.7	33	8.2	0.767

### Antibiotic resistance

The resistance, susceptibility, and relative susceptibility of the strains to beta-lactam antibiotics, including cefotaxime, ampicillin, cefoxitin, aztreonam, amoxicillin-clavulanate, cefalotin, cefixime, imipenem, ceftriaxone, ceftazidime, and non-beta-lactam antibiotics, including ciprofloxacin, tetracycline, ofloxacin, gentamicin, nalidixic acid, co-trimoxazole, and nitrofurantoin were evaluated. The relative frequencies of resistance and susceptibility of ST131 and non-ST131 ESBL-EC strains to beta-lactam and non-beta-lactam antibiotics are shown in Table 6. The frequency of MDR isolates was estimated to be 100%.

**TABLE 6:** Distribution of antibiotic resistance pattern according to ESBL-producing *Escherichia coli* strains isolated from hospitalized patients with urinary tract infections

Antibiotics	Susceptible				Intermediate				Resistant			
	Non-ST131 (*N* = 37)		ST131 (*N* = 70)		Non-ST131 (*N* = 37)		ST131 (*N* = 70)		Non-ST131 (*N* = 37)		ST131 (*N* = 70)	
	No.	%	No.	%	No.	%	No.	%	No.	%	No.	%
Beta-lactam												
Cefotaxime	0	0	0	0	0	0	0	0	37	100	70	100
Ampicillin	0	0	0	0	0	0	0	0	37	100	70	100
Cefoxitin	27	73	41	58.6	4	10.8	16	22.9	6	16.2	13	18.6
Aztreonam	3	8.1	4	5.7	4	10.8	7	10	30	81.1	59	84.3
Amoxicillin-clavulanate	0	0	0	0	0	0	0	0	37	100	70	100
Cefalotin	0	0	0	0	0	0	0	0	37	100	70	100
Cefixime	0	0	0	0	0	0	0	0	37	100	70	100
Imipenem	34	91.9	67	95.7	3	8.1	3	4.3	0	0	0	0
Ceftriaxone	0	0	0	0	0	0	0	0	37	100	70	100
Ceftazidime	2	5.4	3	4.3	6	16.2	6	8.6	29	78.4	61	87.1
Non-beta-lactam												
Ciprofloxacin	5	13.5	13	18.6	3	8.1	2	2.9	29	78.4	55	78.6
Tetracycline	5	13.5	17	24.3	1	2.7	1	1.4	31	83.8	52	74.3
Ofloxacin	7	18.9	17	24.3	0	0	0	0	30	81.1	53	75.7
Gentamicin	23	62.2	45	63.3	1	2.7	3	4.3	13	35.1	22	31.4
Nalidixic acid	3	8.1	4	5.7	0	0	2	2.9	34	91.9	64	91.4
Co-trimoxazole	7	18.9	9	12.9	0	0	0	0	30	81.1	61	87.1
Nitrofurantoin	36	97.3	58	82.9	1	2.7	1	1.4	0	0	11	15.7

According to Fisher’s exact test, there were no significant differences in resistance, susceptibility, or relative susceptibility to any of the beta-lactam antibiotics tested between the ESBL-EC strains in the ST131 and non-ST131 groups (*P* > 0.05). Both groups exhibited 100% resistance to cefotaxime, ampicillin, amoxicillin, cephalothin, cefixime, and ceftriaxone. Additionally, both groups demonstrated the highest susceptibility to the antibiotic imipenem, resulting in a determined resistance level of 0% for this antibiotic in both groups.

Furthermore, Fisher’s exact test for non-beta-lactam antibiotics revealed significant differences in resistance, susceptibility, and relative susceptibility between ESBL-EC strains in the ST131 group and the non-ST131 group, particularly concerning nitrofurantoin (*P* < 0.001). Both groups exhibited high resistance rates to nalidixic acid, at approximately 91%. However, they showed the highest susceptibility to nitrofurantoin, with a notable difference: the resistance rate to nitrofurantoin in the ST131 group was 15.7%, while in the non-ST131 group, the resistance rate was 0% (*P* < 0.001).

## DISCUSSION

*E. coli* ST131 is a major cause of UTIs. Its association with ESBL significantly complicates treatment. In our study, the clinical characteristics and outcomes of the ST131 group were not significantly different from those of the non-ST131 group. It was similar to the study by Zhong et al. (41). Our subtyping analysis revealed that 98.6% (69/70) of the ST131 isolates were fimH30. Among these, 87.1% (*n* = 61), 85.7% (*n* = 60), and 81.4% (*n* = 57) harbored genes encoding *bla*_CTX-M-14_, *bla*_CTX-M-15_, and *bla*_CTX-M-1_, respectively. A study by Muller et al. (5) conducted at a university hospital in France examined the molecular characteristics of ESBL-EC and identified the risk factors associated with *E. coli* ST131 infection or colonization in patients. Every patient who tested positive for ESBL-EC in at least one screening or clinical isolate over two years (2015–2017) was included (*n* = 491). Among all ESBL-EC isolates, 17.5% (*n* = 86) were identified as belonging to the ST131 clone. When these ST131 isolates were subtyped using FimH, 79.1% (68 out of 86) were found to be FimH30. Of the FimH30 isolates, 67.6% (*n* = 46) carried the gene encoding *bla*_CTX-M-15_, 20.6% (*n* = 14) carried *bla*_CTX-M-27_, and 11.8% (*n* = 8) carried *bla*_CTX-M-14_ (5). In a study conducted at a tertiary care hospital in Thailand from March 2015 to June 2017, researchers identified the ST131 clone among clinical *E. coli* isolates. Out of the 58 isolates examined, 59% were from urine samples, and the presence of β-lactamase genes, such as *bla*_CTX-M_, was noted. Although only 31% of the strains were characterized as *E. coli* ST131, a significant 67% were derived from urine samples, and 78% of the patients were over 40 years old. Furthermore, the prevalence of ST131 strains increased over the study period, rising from 10% in 2015 to 30% in 2016, and reaching 73% in 2017 (42).

The incidence of ST131 among 188 ESBL-EC isolates collected in 2015 from AP-HP Beaujon Hospital in Cligny, France, and Lucus Augusti University Hospital in Lugo, Spain, is assessed by Flament-Simon et al. (43). Lucus Augusti reported 33 isolates, accounting for 39.1%, while Beaujon had 46 isolates, which made up 47.9%, with ST131 detection. In total, 109 isolates displayed 57 other sequence types (STs). The most prevalent ESBL identified in both hospitals was *bla*_CTX-M-15_, found in 44.6% of isolates in Lucus Augusti and 50.0% in Beaujon (43). This was the result of a similar research study conducted in Egypt. Out of the 79 isolates that produced urinary ESBL, 54 (68.4%) had the *bla*_CTX-M-15_ gene (41). We found that 39.8% (107/269) of *E. coli* isolates produced ESBL. Among these, 86.9% (*n* = 93), 86.9% (*n* = 93), and 81.3% (*n* = 87) of the coding genes contained *bla*_CTX-M-15_, *bla*_CTX-M-14_, and *bla*_CTX-M-1_, respectively.

In our study, we identified O25b-ST131 subclones in 68 out of 70 urinary ESBL-EC isolates, representing 97.1%. Additionally, O16-ST131 subclones were present in 54 out of 70 isolates, or 77.1%. Similarly, Abdelrahim et al. (44) found that 89 out of 105 *E. coli* isolates in urine, which corresponds to 84.8%, contained the O25b-ST131 clone (44).

Demirci et al. (45) used real-time PCR to gather molecular epidemiological data and identify the presence of the *bla*_CTX-M-15_ and *bla*_CTX-M-1_ genes, as well as the O25b-ST131 clone, in *E. coli* strains from UTIs cases found both in hospitals and in the community. The data reveal that 22%, 73%, and 37% of UTIs are associated with the O25b-ST131 clone, the *bla*_CTX-M-1_ gene, and the *bla*_CTX-M-15_ gene, respectively. When comparing outpatients to inpatients, *E. coli* isolates from inpatients exhibited a significantly higher frequency of the *bla*_CTX-M-1_ gene and the O25b-ST131 clone. Additionally, *E. coli* strains from UTI patients have been shown to carry both the *bla*_CTX-M-1_ and *bla*_CTX-M-15_ genes, along with the O25b-ST131 clone (45). Zhong et al. (41) examined the frequency of ST131 clones in *E. coli* obtained from women with UTIs in central China while also identifying the molecularly defined O25b-ST131 and O16-ST131 serotypes. Out of the 216 *E. coli* isolates, 41 (19.0%) contained ST131 clones. This group included 14 strains of O16-ST131 and 27 strains of O2b-ST131. Aside from the higher prevalence of urinary stones in the ST131 group (43.9% compared to 27.4%, *P* = 0.039), the clinical features and outcomes were generally similar to those in the non-ST131 group. Therefore, the O25b and O16 serotypes were identified as significant *E. coli* ST131 in UTI isolates among Chinese female patients. Monitoring for ST131 infections is particularly important for patients with UTIs who have a history of urinary stones (41).

Additionally, the well-documented component is the rapidly expanding H30-Rx subclone, which includes allele 30 of type 1 fimbrial adhesin gene FimH. However, resistance genes also exist for *bla*_CTX-M_ types that are not associated with the ST131-H30 subclone. Our results indicated that 92.9% (65 out of 70) of the isolates from ST131 were identified as H30-Rx subclones. Hojabri et al. (46) examine the prevalence of the H30/H30-Rx subclones and the O25b/O16 serotypes of the ST131 clone in *E. coli* isolates that cause extraintestinal infections. Their research indicates that the H30 subclone is expanding in Semnan, Iran, and is likely the primary factor driving the successful proliferation of the ST131 clone (46). The study by Karami et al. (47) investigated the ST131 clone in western Sweden. From 123 individuals with ESBL-EC UTIs within a year, all isolates (319/356) were analyzed using O25b- and ST131-H30-Rx. Isolates of O25b-ST131 and ST131-H30-Rx were identified in 44% and 30% of the cases, respectively. The majority was *bla*_CTX-M-1_ (71%). A patient at risk of developing a recurrent urinary tract infection (RUTI) with ESBL-EC should be screened for the MDR H30-Rx subclone early due to its association with multiple recurrences and its high prevalence (47).

Furthermore, our findings indicate that both the ST131 group and the non-ST131 group exhibited the highest susceptibility to imipenem, with a resistance rate of 0%. This observation is consistent with the study by Kim et al. (48), which identified an *E. coli* clinical isolate, BD07372, of sequence type ST131, recovered from a bed sore specimen that showed high-level susceptibility to imipenem (48). Mares et al. (49) conducted a six-month cross-sectional retrospective study to update the rates of resistance and susceptibility among uropathogens essential for optimal treatment. Out of 5,487 patients screened, 524 (9.54%) were female patients who met the inclusion criteria for the study. *E. coli* was identified as the most common pathogen, accounting for 290 cases (55.34%). *E. coli* presented the highest susceptibility rates were observed for fosfomycin (*S* = 96.55%), followed by imipenem (*S* = 93.1%) (49).

### Conclusion

Our study confirmed the predominance of the genes related to the ST131 clone, including O25, O16, H30, and H30-Rx, among ESBL-EC. Among the ST131 clones, the highest frequency belonged to the ST131-H30 (98.6%), and the lowest frequency belonged to the O16-ST131 (77.1%). This present study confirms that the *E. coli* ST131 clone carried *bla*_CTX-M-1_, *bla*_CTX-M-14_, and *bla*_CTX-M-15_ is expanded in our locale, but there was no significant statistical association between the presence of *bla*_CTX-M-1_, *bla*_CTX-M-14_, and *bla*_CTX-M-15_ genes between ESBL-EC ST131 and non-ST131 groups (*P* > 0.05). Combining the resistance genes in the ST131 clone can facilitate its rapid and successful global spread. Continuous monitoring is essential to prevent the spread of resistant clones and ensure effective treatment options.

## Data Availability

The sequences were submitted to the NCBI GenBank nucleotide database under accession numbers PP580026 (*bla*_CTX-M-15_, *E. coli*), PP558342 (*bla*_CTX-M-14_, *E. coli*), PP580027 (*bla*_CTX-M-1_, *E. coli*), PP836284 (O25b-ST131, *E. coli*), PP836285 (O16-ST131, *E. coli*), and PP817070 (ST131-H30, *E. coli*).
